# BnLATE, a Cys2/His2-Type Zinc-Finger Protein, Enhances Silique Shattering Resistance by Negatively Regulating Lignin Accumulation in the Silique Walls of *Brassica napus*

**DOI:** 10.1371/journal.pone.0168046

**Published:** 2017-01-12

**Authors:** Zhangsheng Tao, Yi Huang, Lida Zhang, Xinfa Wang, Guihua Liu, Hanzhong Wang

**Affiliations:** 1 Oil Crops Research Institute, Chinese Academy of Agricultural Sciences; Key Laboratory of Oil Crop Biology and Genetic Improvement, Ministry of Agriculture; Wuhan, Hubei, China; 2 Plant Biotechnology Research Center, School of Agriculture and Biology, Shanghai Jiao Tong University, Shanghai, China; New Mexico State University, UNITED STATES

## Abstract

Silique shattering resistance is one of the most important agricultural traits in oil crop breeding. Seed shedding from siliques prior to and during harvest causes devastating losses in oilseed yield. Lignin biosynthesis in the silique walls is thought to affect silique-shattering resistance in oil crops. Here, we identified and characterized *B*. *napus LATE FLOWERING* (*BnLATE*), which encodes a Cys2/His2-type zinc-finger protein. Heterologous expression of *BnLATE* under the double enhanced CaMV 35S promoter (D35S) in wild-type *Arabidopsis* plants resulted in a marked decrease in lignification in the replum, valve layer (carpel) and dehiscence zone. *pBnLATE*::*GUS* activity was strong in the yellowing silique walls of transgenic lines. Furthermore, the expression pattern of *BnLATE* and the lignin content gradient in the silique walls at 48 days after pollination (DAP) of 73290, a *B*. *napus* silique shattering-resistant line, are similar to those in transgenic *Arabidopsis* lines expressing *BnLATE*. Transcriptome sequencing of the silique walls revealed that genes encoding peroxidases, which polymerize monolignols and lignin in the phenylpropanoid pathway, were down-regulated at least two-fold change in the *D35S*::*BnLATE* transgenic lines. *pBnLATE*::*BnLATE* transgenic lines were further used to identify the function of *BnLATE*, and the results showed that lignification in the carpel and dehiscence zone of yellowing silique also remarkably decreased compared with the wild-type control, the silique shattering-resistance and expression pattern of peroxidase genes are very similar to results with *D35S*::*BnLATE*. These results suggest that *BnLATE* is a negative regulator of lignin biosynthesis in the yellowing silique walls, and promotes silique-shattering resistance in *B*. *napus* through restraining the polymerization of monolignols and lignin.

## Introduction

Rapeseed (*Brassica napus* L.) is widely planted in temperate zones throughout much of northern Europe, northern America, and Asia. Seed shedding from siliques prior to and during harvest is extremely detrimental to rapeseed yield. The resulting loss in yield is estimated as being around 20%, and poor weather conditions before and during harvest can increase losses to 50% [1, 2]. Thus, diminishing silique shattering would increase the proportion of seed recovered during harvesting and have favorable economic consequences. Angiosperms give rise to various types of fruits, and the fruits of most plants are derived from the ovary wall and fertilized ovules. The fruits of more than 3,000 species of *Brassicaceae*, including *Arabidopsis thaliana* and *B*. *napus*, are known as siliques and develop from the fertilized gynoecium. *Arabidopsis* silique development is an excellent model system for studying the mechanisms that determine a plant organ based on the presence of distinctive morphological features [3]. The silique is divided into three major regions, namely the valves (silique walls), replum, and valve margins [4]. The valves encircle and protect the developing seeds, and detach to promote silique dehiscence after seed maturation. The replum, which connects the two silique walls, forms a central ridge that attaches the silique to the plant. The valve margins form at the junction between the valves and the replum and facilitate silique opening through the action of two different cell types. The separation layer or dehiscence zone (DZ) consists of a layer of 2~3 thin-walled cells, which separate the heavily lignified cells of the pericarp edge from the replum. Degradation of the thin-walled cells is followed by detachment of the valves from the replum through cell-cell separation mediated by the secretion of hydrolytic enzymes at about 7~8 weeks after anthesis [5–7]. The lignified margin layer is continuous with the lignified endocarp layer of the valves. Together, these tissues are thought to create a spring-like tension that causes silique dehiscence when the cells contract and the wood fibers of the mature silique tighten during desiccation. Nevertheless, the siliques are resistant to shattering in moist conditions, due to cell swelling and decreased wood fiber elasticity [8]. This suggests that increased silique shattering resistance is associated with different types of cells in the valve, replum, and valve margin and/or a reduction in the degree of lignification of the cells of the mature siliques.

Lignin is critical for the structural integrity of the cell wall, stiffness and strength of the stem [9]. Lignins are derived mainly from three hydroxycinnamyl alcohol monomers, which differ in their degree of methoxylation. These monolignols produce, respectively, p-hydroxyphenyl H, guaiacyl G, and syringyl S phenylpropanoid units when incorporated into the lignin polymer. Lignification is the process by which units are linked together via radical coupling reactions, and plays an important role in the dehiscence of mature fruits [8]. *SHATTERPROOF1* (*SHP1*) and *SHP2* are required for the proper development of fruit valve margins [10]. Loss-function of *SHP1* and *SHP2* results in the absence of the lignified and separation layers, thereby preventing dehiscence. *INDEHISCENT* (*IND*) and *ALCATRAZ* (*ALC*) act downstream of *SHP* [11, 12]. Loss of *IND* leads to defects in the small cells of the separation zone and the adjacent lignified cell layers, and mutation in *ALC* results in the absence of the layer of non-lignified cells at the site of separation. *BREVIPEDICELLUS* (*BP*), a class I *KNOX* gene, is involved in replum development [13]. *BP* appears to regulate lignin biosynthesis, as *bp* mutants display increased lignin deposition following bolting and aberrant lignin deposition in discrete regions of the stem, and plants overexpressing *BP* exhibit decreased lignification [14, 15]. Recently, Crawford, Ditta [16] reported that *NO TRANSMITTING TRACT* (*NTT*), a C_2_H_2_/C_2_HC-type zinc finger protein, plays a role in the differentiation of the transmitting tract in the carpels of *Arabidopsis thaliana*. Chung, Lee [17] showed that an activation-tagged allele of *NTT* (*ntt-3D)* exhibited a silique indehiscence phenotype, and lacked lignified cells in the valve margin. However, these mutants, which were silique shattering-resistant, had reduced valve elongation after fertilization and substantially reduced seed development, especially in the basal part of the fruit due to a lack of coordinated growth of the fruit tissues, resulted in crop failure [10, 12, 17–19].

A new study had revealed a smaller non-lignified separation layer in relatively shatter-resistant *B*. *juncea* relative to *B*. *napus*, which would formation a tension with lignified layer in the silique DZ, and plays a major role in dehiscence. Furthermore, 16 out of 27 genes involved in monolignol biosynthesis pathway in the Brassica cultivars were moderately to highly down-regulated in valve and DZ of *B*. *juncea* compared to *B*. *napus* using microarray analysis [20]. In this study we identified *B*. *napus LATE*, and ectopically expressed this gene in *Arabidopsis*. The silique of *D35S*::*BnLATE* transgenic *Arabidopsis* lines exhibited diminished lignification of the replum, valve layer, and dehiscence zone, leading to enhanced silique shattering resistance, but did not show any other phenotypic variations during silique and seed development. *pBnLATE*::*GUS* was strongly expressed in the replum and yellowing silique walls during development stage 18 as defined by Smyth, Bowman [21], in the transgenic *Arabidopsis* lines. Furthermore, transcriptome analysis revealed that some genes encoding peroxidase, which catalyzes the polymerization of monolignols and lignin in the phenylpropanoid pathway, were down-regulated in *D35S*::*BnLATE* transgenic *Arabidopsis* lines. These observations suggest that *BnLATE* is a key regulator of lignin biosynthesis in *B*. *napus* at specific developmental stages, resulting in enhanced silique shattering resistance in *B*. *napus*.

## Materials and Methods

### Plant growth conditions

The *Arabidopsis thaliana* Col-0 ecotype was used as wild type in this study. Seeds of Col-0, empty vector control (EV) lines, and transgenic lines expressing *BnLATE* were sown in 0.5-L pots containing a mixture of soils (Peilei™, Zhenjiang, China), pre-incubated for 3 days in darkness at 4°C, and then transferred to a growth room at 21°C, under a 16/8 h light/dark photoperiod, and ~150 μE.m^-2.^s^-1^ light intensity at Oil Crops Research Institute of Chinese Academy of Agricultural Sciences (OCRI-CAAS) (Wuhan, China).

*B*. *napus* accessions Zhongshuang 11(ZS11) and 73290 were previously developed in OCRI-CAAS and sown at the experimental station of OCRI-CAAS on September 27, 2014, under normal growth conditions. The owner provided full access permission to the experimental site. The field trails did not range over any protected or endangered species, and no vertebrates were involved in this study. Individual plants of each accession without obvious phenotypic differences were reserved through eliminating potential false type at several development stages.

### BnLATE isolation and expression vector construction

Using a Genome Walking Kit (Cat: D316, TAKARA) with specific primers wlate-1, -2, and -3-r, an 849-bp fragment was amplified from the genome of *B*. *napus* ZS11 following the manufacturer’s instructions, and the *BnLATE* promoter sequence was subsequently isolated using primers plate-f/r and used to generate the *pBnLATE*::*GUS* construct. For the *pD1301S*::*BnLATE* construct, a full-length coding sequence (592 bp) was amplified from *B*. *napus* ZS11 using primers glate-f/r. Thirty cycles of PCR were carried out under the following program: 94°C for 30 s, 58°C for 30 s, and 72°C for 40 s. The amplicons were cloned using TA-overhangs into the pMD18-T vector (TAKARA). The integrity of the amplicons was confirmed by sequencing using M13 primers. These sequences were transferred to the expression vectors, pD1301S and pC1301GT, to construct expression plasmids *pD1301S*::*BnLATE* and *pBnLATE*::*GUS*, which were further confirmed by PCR using a primer pair of the cauliflower mosaic virus 35S constitutive promoter (35s-f) and glate-r and of pC1301GT (cgt-r) and plate-f, respectively. All primers for target genes used in this study are listed in S3 Table.

### Generation of transgenic plants

*Agrobacterium tumefaciens* strain GV3101 competent cells were prepared and transformed with *pD1301S*::*BnLATE* and *pBnLATE*::*GUS*. Then *GV3101-BnLATE* and *GV3101-pBnLATE*::*GUS* were transferred into Col-0 using the floral dip method [22]. Transformants were germinated and screened on Murashige and Skoog medium containing 50 mg/L hygromycin B (Cat.10843555001, ROCHE), followed by PCR verification using the glate-f/r and plate-f/r primers, respectively. Positive seedlings (T_0_) were transferred to 0.5-L pots containing a mixture of soils (Peilei™, Zhenjiang, China) and maintained in a growth room as above.

### Validation of *BnLATE* using real-time PCR

The silique walls were collected at 15 days after pollination (DAP), namely stage 18 as defined by Smyth, Bowman [21], when the valve had completely elongated and the valve edges and endocarp had lignified, from the Col-0 and transgenic *Arabidopsis* lines (EV, L7, L8, L21). Total RNA was extracted using an RNAprep Pure Plant Kit (Cat.DP432, TIANGEN, China), according to the manufacturer’s protocol. *AtActin2* (AT3G18780) was used as a reference for normalizing the transcript level of target genes in each *Arabidopsis* sample. Oligonucleotide primers for amplifying reference and target genes by real-time PCR were designed using Primer Premier 5 (http://www.premierbiosoft.com), and primer sequences are shown in S3 Table.

About 3.0 μg of total RNA for each sample was reverse transcribed into first-strand cDNA using Oligo (dT_18_) and 200 U M-MLV reverse transcriptase (Cat.M1701, Promega) under the following conditions: 70°C for 5 min, ice-bath for 5 min, and 42°C for 60 min. Real-time assays were conducted for all target and respective reference genes based on three biological and two technical replicates using a CFX96 Real-time PCR Detection System (Bio-Rad). iTaq^TM^ Universal SYBR^®^ Green Supermix (Cat.172-5121, Bio-Rad) was used in a final volume of 20.0 μL containing 10.0 μL of 2×SYBR Green Master Mix, 0.5 mM of each primer, and 2.0 μL of cDNA. PCR conditions were as follows: 95°C for 2 min, followed by 40 cycles of 95°C for 15 s, 58°C for 15 s, 72°C for 25 s, and 25°C for 30 s. Gene expression and statistical analyses were performed using Bio-Rad CFX Manager (Version 2.1, Bio-Rad) and Excel 2010 (Microsoft Corporation, USA).

### Semi-quantitative RT-PCR analysis of BnLATE

Total RNA was isolated from the silique walls of rapeseed 73290 using an RNAprep Pure Plant Kit (Cat.DP432, TIANGEN, China). The glate-f/r primers were designed based on the *BnLATE* coding sequence and used to amplify *BnLATE* in a semi-quantitative RT-PCR analysis. *BnActin2* (with primers BnActin2-f/r) was used as a reference to evaluate the relative amount of *BnLATE* mRNA in each sample. RT-PCR was carried out using an Easy Taq DNA Polymerase Kit (TransGen) according to the manufacturer’s protocol. Briefly, samples were incubated at 70°C for 5 min, in an ice-bath for 5 min, and then at 42°C for 60 min. PCR was performed at 94°C for 3 min followed by 33 cycles of 94°C for 30 s, 58°C for 30 s, and 72°C for 40 s. Primer sequences are shown in S3 Table.

### GUS staining

Organs of transgenic *pBnLATE*::*GUS* plants were collected and submerged in GUS buffer as previously described [23]. All samples were infiltrated thrice (for 5 min) under a vacuum, incubated at 37°C for 1 to 2 h, and then washed thrice with 70% ethanol. All samples were observed and photographed under white light.

### Phylogenetic analysis

Ten well-known C_2_H_2_ zinc-finger family genes from *Arabidopsis* and BnLATE were subjected to phylogenetic analysis using the neighbor-joining (NJ) method in MEGA4 with default settings [24]. The bootstrap test was carried out with 1,000 replicates.

### Histological analysis

For microscopy analysis of the anatomical features of the siliques of the *D35S*::*BnLATE* transgenic lines, controls (wild-type Col-0 and EV), and *B*. *napus* accessions (73290), fresh plant tissues were collected and fixed in FAA buffer (4% formaldehyde, 50% ethanol, 5% acetic acid), dehydrated in an ethanol series from 70% to 100%, and infiltrated with xylene followed by paraffin. Blocks were cast following a general embedding procedure. The specimen block was trimmed to give a trapezoid shape and then cut with a microtome (RM2016, Leica, 4 μm thickness) until the internal anatomy of the specimen was exposed at the region of interest [25]. Samples were stained using Safranin/Fast Green Staining Kits (Cat.BL1081, Bioyear-Biotechnology), according to the procedure of Lenser and Theissen [26]. The stained surfaces of specimen blocks were treated with immersion oil and analyzed using a fluorescence digital inverted microscope (IX71, Olympus).

### Total lignin assay

Silique walls were collected from transgenic lines (L7, L8, and L21), Col-0, and EV at 15 DAP as well as from the *B*. *napus* accessions 73290 at 12, 24, 36, 48, and 60 DAP, respectively. Total lignin content was measured in the silique walls using a two-step acid hydrolysis method as previously described [27]. Lignin includes acid-insoluble and acid-soluble types. The acid-insoluble lignin (AIL) was calculated gravimetrically after correction for ash, and the acid-soluble lignin (ASL) was measured by UV spectroscopy. The following formula was used:

AIL(%)=(W2−W3)×100/W1,

where W1 is the calculated weight of the sample, W2 is the weight of the crucible and dry residue weight after acid dissolution, and W3 is the weight of the crucible and ash.

ASL(%)=(A×D×V/1000×K×W1)×100,

where W1 is the calculated weight of the sample, A is the absorption value, D is the dilution ratio of the sample, and K (the absorptivity constant) = 110 L/g/cm.

Totallignin(%)=ASL%+AIL%.

All experiments were carried out in triplicate.

### Silique shattering resistance index (SRI) assay

Mature siliques of 73290 were collected for measurement of silique shattering resistance by a random impact test [28]. Briefly, 20 siliques were reserved in an air-tighten bag overnight after incubation at 80°C for 30 minutes to equilibrate moisture, then continuous record cracked siliques after 2-min intervals of shaking for five times on a shaker with 12 steel balls (diameter 13 mm) at 252, 280 and 313 revolutions per minute (rpm) in a cylindrical container, which was 19 cm in diameter and 14 cm in height. The broken pods were removed from the container after each counting. Five plants of each variety were tested and pods were collected randomly from each whole plant. The pod shatter resistance index was calculated using the following equation:

$$(SRI)=1-\sum_{i=1}^{10}xi\times (11-i)/200$$

Where *X*_*i*_ is the number of cracked siliques in time i^th^, 1 ≤ i ≤ 10.

All experiments were carried out in triplicate.

### Transcriptomic analysis by RNA-seq

Trancriptomic analysis of the silique walls of Col-0 and *BnLATE* transgenic *Arabidopsis* line L8 was executed using RNA-Seq with an Illumina HiSeqTM 2000 platform (Sangon Biotech, Shanghai, China). RNA-Seq data were processed as previously described [29]. To identify differentially expressed genes (DEGs), a previously described method was used [30]. Finally, FDR ≤ 0.05 and an absolute value of Log_2_ ratio ≥ 1 were selected as the significance threshold of differential gene expression in the siliques of wild type Col-0 and transgenic line L8. Gene ontology and pathway mapping analysis of the identified DEGs were performed using MapMan and the KEGG database.

### Anthocyanin content determination

To estimate the total anthocyanin content, 15-DAP siliques were dried and ground into fine powder in liquid N_2_ and extracted with acidic methanol (0.1% HCl, v/v) for 18 h at room temperature. After extraction, samples were centrifuged for 20 mins at 5,000 g. Anthocyanin in the supernatant was determined spectrophotometrically through recording *A*_*530*_ and *A*_*657*_. Quantification of anthocyanin was carried out as follows: anthocyanin = (*A*_530_ − 0.25 × *A*_657_)M^−1^, where M is the weight of the plant material used in the analysis [31, 32].

### Complementation test

The *pBnLATE*::*BnLATE* construct containing a 1358 bp fragment from ZS11, including the full-length ORF (513 bp) and the putative promoter sequence (845 bp) of *BnLATE*, was obtained using primers glate2-f/r and plate2-f/r, and inserted into pDX2181 [33]. The resulting construct was introduced into *Agrobacterium tumefaciens* strain GV3101 and transformed the Col-0 plants following *Agrobacterium*-mediated transformation procedure [22]. All primers were listed in S3 Table.

## Results

### Isolation and characterization of BnLATE in *B*. *napus*

**Fig 1:**
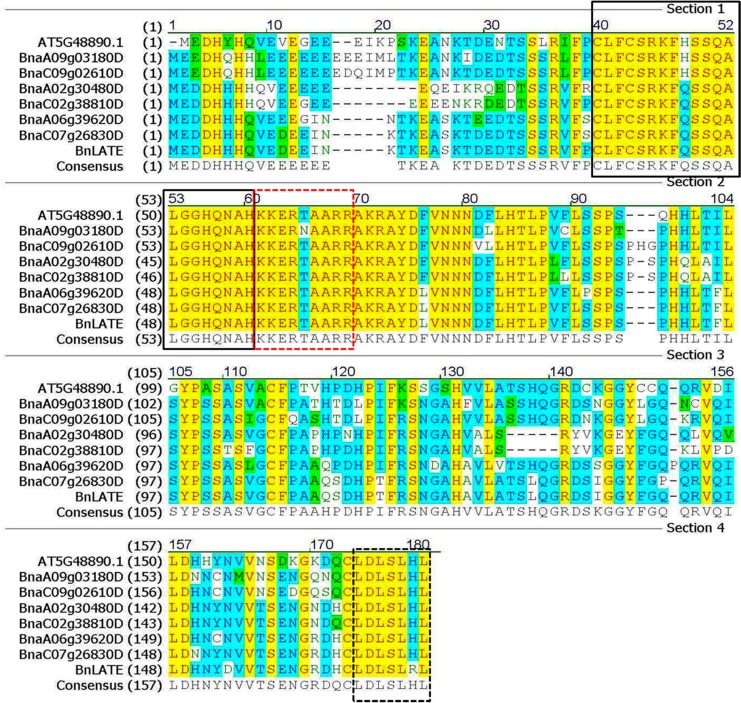
Amino acid sequence alignment of BnLATE and *Arabidopsis* LATE. The C_2_H_2_ zinc-finger domain, LDLXL domain, and B-box indicated by solid black, dotted black, and dotted red boxes, respectively. Numbers in parentheses indicate the amino acid position in each protein.

Using a *Brassica* 90K Oligo Array (Combimatrix), we analyzed the transcriptomic profiles of the main inflorescences of two *B*. *napus* accessions, ZS11 and 73290, and identified a differentially expressed probe (MI7092; the expression ratio of ZS11 vs. 73290 was 4.4) corresponding to a cDNA (EST, GenBank accession No. EE445734) with homology to *A*. *thaliana LATE FLOWERING* (*AtLATE*) [34]. The full-length ORF (513 bp) of this probe in both ZS11 and 73290 were isolated and sequenced, and both sequences were found to be identical to each other. With 76% amino acid identity to AtLATE (Fig 1), this ORF was thereby named *BnLATE*. Moreover, sequence comparison showed that rapeseed contained six *BnLATE* homologues named *BnaA02g30480D*, *BnaA06g39620D*, *BnaA09g03180D*, *BnaC02g38810D*, *BnaC07g26830D*, and *BnaC09g02610D*, which were located in the A genome (A02, A06, and A09 chromosomes) and the C genome (C02, C07, and C09 chromosomes) respectively, based on the genome sequence of rapeseed (www.ncbi.nlm.nih.gov/assembly/GCA_000686985.1/) (Fig 1). In which *BnLATE* had 99% identity at nucleic acid level to *BnaC07g26830D*, and was chosen for further research.

**Fig 2:**
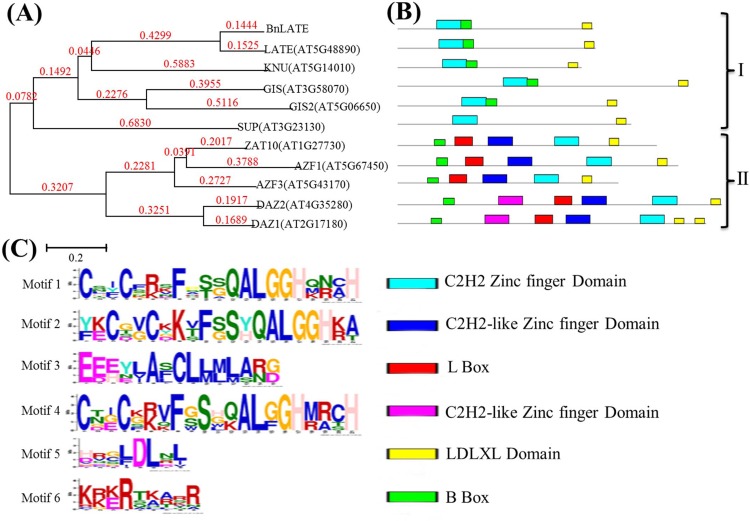
Molecular phylogenetic tree and schematic functional domains of the Cys2/His2-type zinc-finger proteins. (A) Molecular phylogeny of the Cys2/His2 type zinc-finger proteins. *BnLATE* and *AtLATE* clustered into a group. Unrooted neighbor-joining tree of the C-terminal end of amino acid sequences encompassing the predicted mature domain. Branch lengths scaled to the number of amino acid changes indicated on the scale bar. (B) Schematic functional domains of the Cys2/His2 type zinc-finger proteins. I and II denote two clades. Conserved motifs identified within amino acid sequences using the online MEME (http://meme.nbcr.net/meme/cgi-bin/meme.cgi). The motifs and their corresponding positons were represented by different colors of square. The parameters of MEME were set as following: optimum width 10–100 amino acids, the max number of any repeated motifs was set at 25. (C) Conserved motif models identified with MEME procedure among members of LATE family from *B*. *napus* and *Arabidopsis*. Motifs 1, 2, and 4 are zinc finger domain, motifs 3, 5, and 6 are L-box, EAR-like domain, and B-box respectively.

In *silico* analysis indicated that *BnLATE* encodes an intron-less zinc-finger protein transcription factor that consists of 171 amino acids, including three conserved domains, namely a C_2_H_2_ zinc-finger domain, B-box domain, and LDLXL domain (Fig 1). The evolutionary relationship between *BnLATE* and 10 well-known zinc-finger family genes in *Arabidopsis* was evaluated by phylogenetic analysis using the unrooted neighbor-joining (NJ) method, and these genes were grouped into two distinct clades (Fig 2A). The number of zinc-finger domains differed in each gene, and three conserved domains detected in BnLATE were identified in all clade I and II proteins (Fig 2B). Besides the three conserved domains, two other C_2_H_2_-like zinc-finger domains (consensus sequence CX_2_CXKXFXSXQALGGHX_3_H, CX_2_CX_3_FXSX2ALGGHX_3_H, respectively) and an L-box domain were only present in the N-terminal of clade II proteins (Fig 2B and 2C), and the functions were unknown [35, 36].

### *BnLATE* is specifically expressed in the yellowing silique wall

To analyze the temporal and spatial expression pattern of *BnLATE* in *B*. *napus*, we isolated and sequenced the *BnLATE* promoters (849 bp) from 73290 using genome walking PCR. Within the 849 bp sequences, a TATA box and a CAAT box, which were the core function domains of promoter, were found through bioinformatics analysis (http://bioinformatics.psb.ugent.be/webtools/ plantcare/html/) (S1 Fig). We then examined the tissue-specific activity of the *BnLATE* promoter from ZS11 by generating the *pBnLATE*::*GUS* construct and transforming it into *Arabidopsis* Col-0 plants.

**Fig 3:**
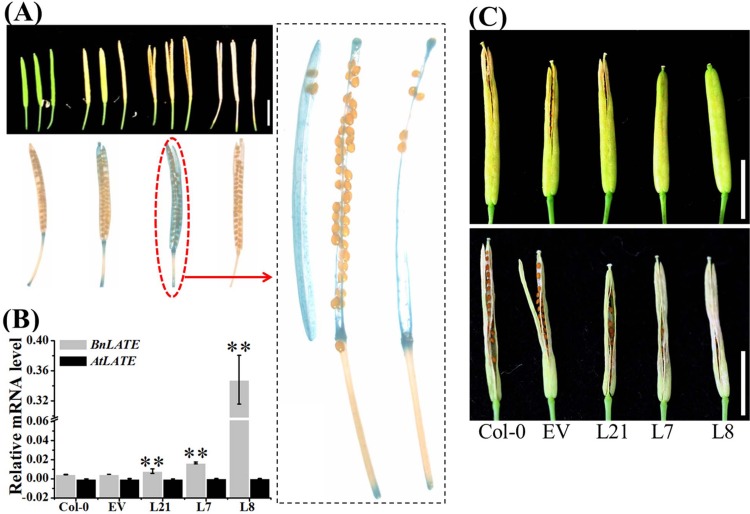
Phenotypic variations caused by *BnLATE* overexpression in *Arabidopsis*. (A) GUS activity was detectable in silique of *pBnLATE*::*GUS* transgenic Arabidopsis. The dotted black line boxes showed that amplification of silique in the red line boxes. Bar, 0.50 cm. (B) Expression level of *BnLATE* in the silique of *D35S-BnLATE transgenic* lines was revealed by Real-time PCR. Expression level of *BnLATE* and *AtLATE* was determined relative to that of the internal control *β-AtACTIN2* (AT3G18780), multiplied by 10. The experiment was performed in triplicate. Significance was determined with Student’s *t*-test. Asterisks above the columns indicate the significant difference compared to Col-0 and EV. * p< 0.05, ** P< 0.01. (C) Representative images of yellowed siliques of controls Col-0, EV and *D35S-BnLATE* transgenic lines (T5). Bar, 0.50 cm

Ten independent positive *pBnLATE*::*GUS* transgenic *Arabidopsis* lines were obtained and grown under long day (LD) condition, and the GUS activity exhibited tissue-specific expression throughout development. In young transgenic seedlings, GUS activity was clearly visible in the roots, shoots, and leaves. Whereas during flowering, it was mainly confined to the flower (S2 Fig). Significantly, GUS was particularly active in the transgenic silique walls and replum of yellowing siliques (Fig 3A).

### Overexpression of *BnLATE* in *Arabidopsis* reduced the lignin content of the silique

Weingartner, Subert [37] reported that no T-DNA insertion lines of *AtLATE* existed and *LATE-RNAi* lines did not show any phenotypic variation at any developmental stage, suggesting that the loss-of-function approach is not feasible for examining the in vivo function of *AtLATE*. However, overexpression of *AtLATE* in *Arabidopsis* leads to delayed flowering, inflorescence growth repression, and formation of sterile and incomplete flowers [37]. Given the success of gain-of-function approaches in examining *AtLATE*, we decided to explore the physiological role of *BnLATE* by ectopical expressing in *Arabidopsis*.

We isolated the full-length cDNA of *BnLATE*, fused it downstream of the double CaMV 35S promoter (D35S) in a vector, and transformed the resulting vector into *Arabidopsis* wild-type Col-0 plants using the floral dip method [22]. We used the D35S empty vector (EV) as the control. After hygromycin B selection, 22 independent *D35S*::*BnLATE* transgenic *Arabidopsis* lines (L1-L22) were recovered, and 15 out of 22 (68.2%) transgenic lines showed enhanced silique shattering resistance compared with the Col-0 and EV plants, suggesting that overexpression of *BnLATE* in *Arabidopsis* could cause variation on dehiscence properties of siliques.

**Fig 4:**
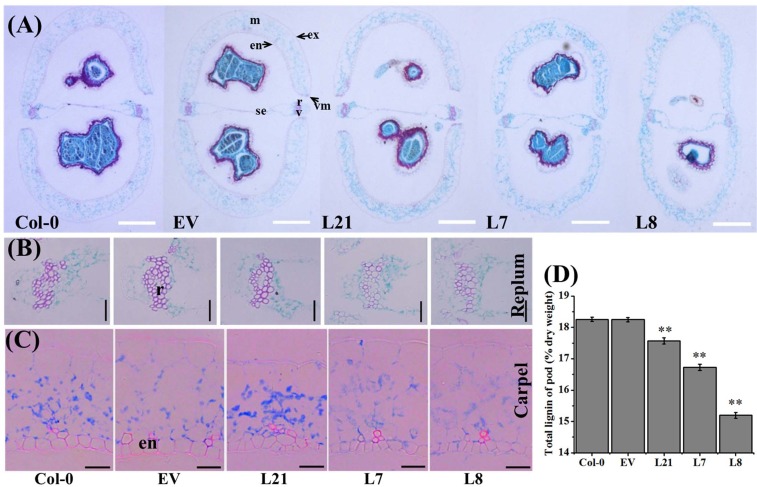
Histological analysis of 15-DAP silique cross-sections of *D35S*::*BnLATE* transgenic *Arabidopsis* lines. **DAP: days after pollination.** (A) Microscopy observations of cross-sections of *D35S*::*BnLATE* transgenic *Arabidopsis* siliques stained with Safranin/fast green. A representative result of five independent experiments is shown. Bars, 100 μm. (B) Microscopy observations of the stained replum. Bar, 50 μm. (C) Microscopy observations of the stained carpel. Bar, 10 μm. (D) Determination lignin content in the 15-DAP siliques of *D35S*::*BnLATE* transgenic *Arabidopsis* lines. The experiment was performed in triplicate, and results represent the mean ± S.D. Significance was determined with Student’s *t*-test. Asterisks above the columns indicate the significant difference compared to Col-0 and EV. * p< 0.05, ** p< 0.01. Abbreviations: en, endocarp; ex, exocarp; m, mesocarp; r, replum; se, septum; sl, separation layer; v, vascular bundle; vm, valve margin.

To confirm that this phenotypic variation in the *BnLATE* transgenic *Arabidopsis* lines was caused by the ectopic expression of *BnLATE*, we first examined the expression level of *BnLATE* in 9-day-old plants of the transgenic lines and the controls Col-0 and EV. Interestingly, the expression of *BnLATE* in transgenic lines was 0.007 times over that of the internal control *ACTIN2* (data not shown), as the levels of *BnLATE* increased, so did the proportion of siliques exhibiting shattering resistance. Since silique shattering resistance was negatively associated with the degree of lignification in the valves, replum, and valve margin cells [8], Col-0, EV, and three representative *BnLATE* transgenic lines (L21, L7, and L8), with increasing levels of *BnLATE* expression in the siliques (Fig 3B) and silique shattering resistance (Table 1 and Fig 3C), were chosen to evaluate the lignin density in the silique wall. We determined the degree of lignification in morphologically identical siliques at 15 DAP in dissected tissues as previously described [25, 26]. Compared with Col-0 and EV, staining color of Safranin/Fast Green was weaker in the replum of siliques of transgenic lines L8 and L7, but not of L21 (Fig 4A and 4B), was well as reduced in the carpels (endocarp b) of transgenic lines L8, L7, and L21 (Fig 4A and 4C). No significant differences were observed in the structure of the valve margin cells, which consist of a layer of lignified cells and a separation layer of small cells, in the replum region, and the valve layer cells.

**Table 1:** Percentage of *D35S*::*BnLATE* transgenic *Arabidopsis*, Col-0 and EV plants exhibiting silique shattering resistance.

Lines	Average percentage of plants with All-, Half- and Non-shattered siliques*		
	All	Half	Non
Col-0	0.49±0.06	0.31±0.07	0.19±0.05
EV	0.48±0.06	0.36±0.05	0.18±0.04
L21	0.43±0.03	0.40±0.06	0.17±0.04
	(*P = 0*.*03*)	(*P = 0*.*03*)	(*P = 0*.*42*)
L7	0.21±0.05	0.48±0.07	0.32±0.05
	(*P = 5*.*79×10*^*−10*^)	(*P = 6*.*98×10*^*−5*^)	(*P = 4*.*43×10*^*−6*^)
L8	0.21±0.07	0.43±0.04	0.37±0.05
	(*P = 3*.*65×10*^*−9*^)	(*P = 0*.*001*)	(*P = 6*.*09×10*^*−8*^)

*The experiment was performed in triplicate (30 siliques each). The significant difference compared to Col-0 and EV was determined with Student’s t-test. p value was shown in the parenthesis, significance was bolded.

Furthermore, we examined the lignin content in the silique walls at 15 DAP of transgenic lines L21, L7 and L8 as well as the controls Col-0 and EV. The total lignin content in the silique wall of three transgenic lines was significantly lower than that of the controls (Fig 4D), while that in the transgenic lines was progressively less in the transgenic lines L21, L7 to L8, which exhibited increasing levels of *BnLATE* expression (Fig 3B). In other words, the amount of total lignin in silique walls of the controls (Col-0, EV) and transgenic lines (L21, L7, and L8) was inversely associated with the level of *BnLATE* expression in the siliques of these lines.

Taken together, heterologous expression of *BnLATE* in the silique wall reduced the degree of lignification in the replum, valve margin, and valve layer cells in *Arabidopsis*, resulting in enhanced plasticity of yellowing siliques.

### Endogenous expression variation of *BnLATE* in *B*. *napus*

Since the heterologous expression of *BnLATE* in *Arabidopsis* resulted in reduced lignification of the silique walls and enhanced silique shattering resistance, whether had the same expression pattern of *BnLATE* and lignification changes of the silique wall in rapeseed variety 73290, a *B*. *napus* silique shattering-resistant line (shattering resistance index (SRI)> 0.7, as an indicator of silique shattering resistance),due to much lower *BnLATE* expression in inflorescence of 73290 revealed by microarray analysis [34].

**Fig 5:**
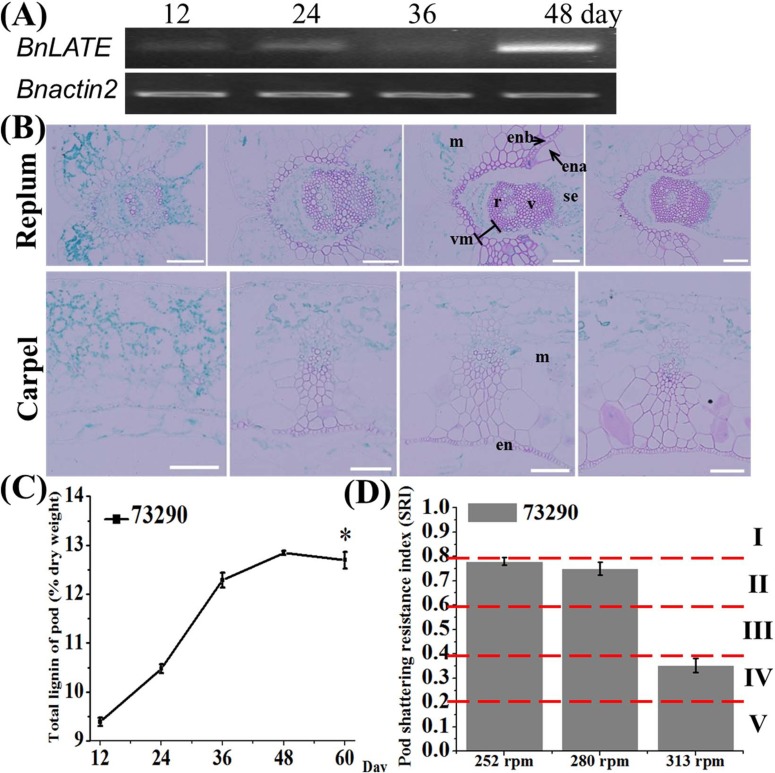
Histological analysis and expression level of *BnLATE* in silique of *B*. *napus* accessions 73290. (A) *BnLATE* expression level was examined by RT-PCR. *β-BnACTIN2* was used for standardization of the template concentration. (B) Microscopic observations of the replum and carpel stained by safranin/fast green. One representation of five independent experiments is shown. 12, 24, 36, and 48 DAP from left to right. Bars, 100 μm. (C) Determination of lignin content in the silique wall of 73290 at 12, 24, 36, 48, and 60 DAP. (D) Silique shattering-resistance index (SRI) of the mature silique of 73290 was determinated by a random impact test. The test was performed in triplicate. Significance was defined with Student’s *t*-test. I, II, III, IV and V showed that five SRI from high to low. Abbreviations: en, endocarp; ena, endocarp a; enb, endocarp b; m, mesocarp; r, replum; se, septum; sl, separation layer; v, vascular bundle; vm, valve margin.

To test this hypothesis, we examined *BnLATE* expression in the silique walls of 73290 at 12, 24, 36, and 48 DAP using semi-quantitative RT-PCR. *BnLATE* was remarkably detectable at 48 DAP, but was very weak at the three earlier points (Fig 5A). The expression diagram was semblable with the pattern of *pBnLATE*::*GUS* activity in *Arabidopsis* (Fig 3A). In addition, siliques at the above four developmental stages were harvested and fixed with FAA buffer, dissection and staining with Safranin/Fast Green buffer was performed as previous description [25, 26], lignin in the silique walls stained red. The intensity of staining gradually increased in the replum, valve margin, and valve layer of 73290 from 12 to 36 DAP, but the intensity of staining at 48 DAP was a little lower than at 36 DAP (Fig 5B). These results were further proved to be reliable at the lignin content measured in the silique walls of 73290 at 12, 24, 36, 48, and 60 DAP (Fig 5C). Furthermore, the silique shattering resistance index (SRI) was measured in mature siliques of 73290 using a previously described random impact test [28]. As shown in Fig 5D, the SRI of 73290 was as high as 0.78 and 0.75 at 252 and 280 revolutions per minute (rpm), respectively, and under the extreme rpm (313) still with 0.35. These results indicate that *BnLATE* activity was important for proper lignification in the replum, valve margin, and valve layer of the yellowing silique wall, and thus affects the silique shattering resistance index of the mature silique in *B*. *napus*.

### Transcriptomic analysis of BnLATE-regulated pathways

**Fig 6:**
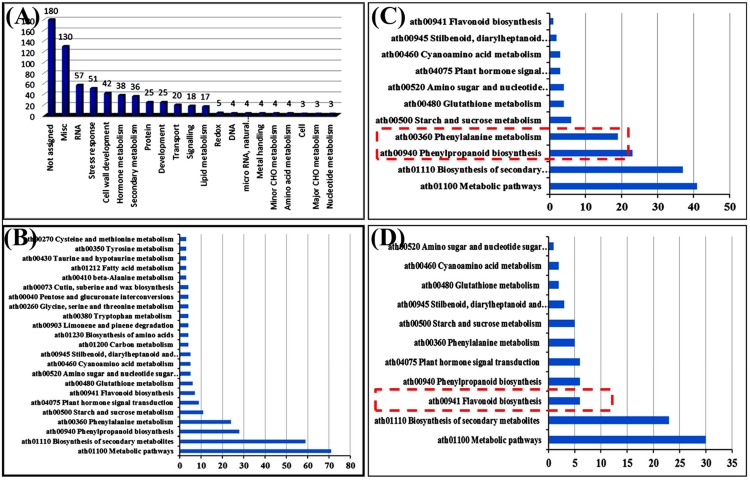
Differentially expressed genes in overexpressing *BnLATE Arabidopsis* lines. (A) Functional categories of differentially expressed genes (DEGs) in a D35S::*BnLATE* transgenic *Arabidopsis* line. (B) Mapped DEGs on KEGG biological pathways. (C) Down-regulated DEGs encoding enzymes in a *D35S*::*BnLATE* transgenic *Arabidopsis* line L8. Red dashed box indicates the preferred pathways. (D) Up-regulated DEGs encoding enzymes in a *D35S*::*BnLATE* transgenic *Arabidopsis* line L8. Red dashed box indicates the preferred pathways.

To investigate the genome-wide effects of *BnLATE* overexpression on transcription, we performed transcriptome sequencing 15-DAP siliques of the D35S::*BnLATE* transgenic line L8 and control Col-0 plants. A total of 33854 genes were expressed both in L8 and Col-0. Using a combined criterion of 2-fold or greater change and a false discovery rate (FDR) of <0.05, 385 and 291 genes were up- and down-regulated in transgenic line L8 vs. Col-0, respectively (S1 Table). These differentially expressed genes (DEGs) in the *BnLATE* overexpression line L8 with respect to their roles were grouped based on TAIR10. A total of 21 categories harboring at least three or more genes were classified and 14 categories were highly enriched with strong confidence levels (p<0.05), including categories such as cell wall development, secondary metabolism, and hormone metabolism (Fig 6A). Interestingly, 119 of the 676 DEGs encoding enzymes were assigned into 23 pathways harboring at least three or more DEGs according to KEGG analysis (Fig 6B and S2 Table). About 53.8% (64 out of 119) of the DEGs were down-regulated in L8 and that these genes were mainly involved in phenylpropanoid biosynthesis, phenylalanine metabolism, starch and sucrose metabolic pathways (Fig 6C). The remaining 46.2% of DEGs were up-regulated in L8 and were mainly demanded for flavonoid biosynthesis and plant hormone signal transduction (Fig 6D).

### Expression of genes related to phenylpropanoid biosynthesis pathway

**Fig 7:**
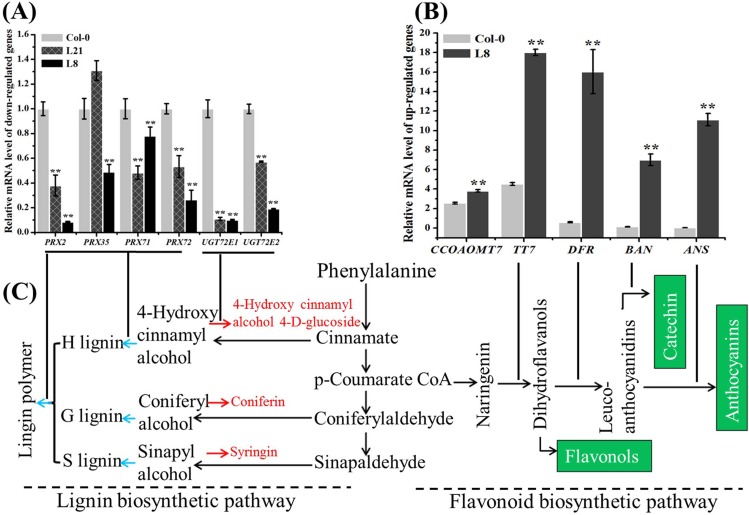
Schematic diagram of lignification and the expression pattern of genes involved in lignin biosynthesis in wild-type Col-0 and *D35S*::*BnLATE* transgenic *Arabidopsis* lines. (A) Validation by real-time PCR of representative DEGs that are down-regulated in the siliques of *D35S*::*BnLATE* transgenic *Arabidopsis* lines (L21 and L8) and Col-0. Results represent the mean ± S.D from three replicates. (B) Validation by real-time PCR of representative DEGs that are up-regulated in the siliques of *D35S*::*BnLATE* transgenic *Arabidopsis* lines (L8 shown) and Col-0. The expression level was determined relative to that of the internal control, *β-AtACTIN2* (AT3g18780). The experiment was performed in triplicate, and results represent the mean ± S.D. Significance was determined using Student’s *t*-test. Asterisks indicate significant differences between Col-0 and EV. * p< 0.05, ** p< 0.01. (C) A schematic model of lignin and flavonoid biosynthesis in *Arabidopsis*.

Most of the structural genes of the phenylpropanoid pathway were detected by RNA-seq both in the transgenic line L8 and control Col-0. Among those DEGs involved in phenylpropanoid biosynthesis, 19 encoding peroxidase (EC-1.11.1.7), two encoding UDP-glucosyl transferase (EC-2.4.1.111), and two encoding β-glucosidase (EC-3.2.1.21) were down-regulated in the L8 transgenic line (Table 2). The 19 down-regulated DEGs encoding peroxidase (EC-1.11.1.7), were related to the final step of lignin biosynthesis, in which 4-hydroxy cinnamyl, coniferyl, and sinapyl alcohols are respectively coupled to polymerizing *p*-hydroxyphenyl (H), guaiacyl (G), and syringyl (S) units [38], resulting in lignin polymers (Fig 7C). Four (AT1G05250, AT3G21770, AT5G64120 and AT5G66390) of the 19 down-regulated DEGs encoding cationic cell wall-bound peroxidase homologs (PRX2, PRXR9, PRX71 and PRX72), had been verified to oxidize monolignols efficiently and lignin oligomers directly, which was highly consistent with previous analyses of the syringyl unit and lignin contents in *prx2*, *prx71*, and *prx72* mutants with *Arabidopsis* Col-0 background [15, 38–41] (Table 2). In addition, two DEGs (AT3G50740 and AT5G66690) encoding UDP-glucosyl transferases (UGT72E1 and UGT72E2, EC-2.4.1.111) were down-regulated in the transgenic line L8. UGT72E1 and UGT72E2 encode UDPG coniferyl alcohol glucosyltransferases that specifically glucosylate sinapyl- and coniferyl-aldehydes, which are thought to be involved in lignin metabolism. Previous studies [42, 43] reported that the level of coniferyl alcohol 4-*O*-glucoside and sinapyl alcohol 4-*O*-glucoside in the *UGT72E2-RNAi* knockdown line was two-fold lower than in the wild type. Using real-time PCR, we validated the expression pattern of the above-mentioned genes in the siliques at 15 DAP of the transgenic *Arabidopsis* lines L8, L21 and the control Col-0, which were highly consistent with that of RNA-seq analysis (Fig 7A and Table 2), suggesting that *BnLATE* overexpression strongly diminished lignin biosynthesis in the silique wall by lowering the expression level of genes encoding peroxidases.

**Table 2:** Differentially expressed genes involved in the lignin polymerization.

Gene ID	Col-0 silique	*BnLATE* silique	Fold change (log2 ratio)	EC code	Description
Down-regulated genes					
AT1G05240	10.644	3.691	-1.528	EC-1.11.1.7	Peroxidase superfamily protein
AT1G05250	9.311	3.854	-1.273	EC-1.11.1.7	PRX2; peroxidase that is involved in the lignification of cell walls
AT1G30870	11.903	3.443	-1.790	EC-1.11.1.7	Peroxidase superfamily protein
AT1G66270	37.119	12.545	-1.565	EC-3.2.1.21	BGLU21; β-glucosidase that has a high level of activity against the naturally occurring secondary metabolite scopolin.
AT1G66280	17.815	8.074	-1.142	EC-3.2.1.21	BGLU22; β-glucosidase activity, hydrolase activity, hydrolyzing O-glycosyl compounds
AT2G35380	8.565	2.753	-1.637	EC-1.11.1.7	Peroxidase superfamily protein
AT3G09260	472.527	192.648	-1.294	EC-1.11.1.7	PYK10; glycosyl hydrolase superfamily protein
AT3G21770	13.917	6.050	-1.202	EC-1.11.1.7	PRX13; peroxidase superfamily protein
AT3G49960	9.161	3.815	-1.264	EC-1.11.1.7	PRX35; Peroxidase superfamily protein
AT3G50740	47.126	23.082	-1.030	EC-2.4.1.111	UGT72E1; UDP-glucosyl transferase 72E1
AT4G11290	8.752	3.438	-1.348	EC-1.11.1.7	Peroxidase superfamily protein
AT4G26010	15.567	4.326	-1.847	EC-1.11.1.7	Peroxidase superfamily protein
AT4G30170	31.607	14.716	-1.103	EC-1.11.1.7	Peroxidase family protein
AT4G36430	6.687	2.271	-1.558	EC-1.11.1.7	Peroxidase superfamily protein
AT5G17820	35.452	14.692	-1.271	EC-1.11.1.7	Peroxidase superfamily protein
AT5G39580	10.227	4.004	-1.353	EC-1.11.1.7	Peroxidase superfamily protein
AT5G51890	10.930	5.014	-1.124	EC-1.11.1.7	PRX66; Peroxidase involved in the lignification of tracheary elements (TE) in roots
AT5G64100	40.566	15.383	-1.399	EC-1.11.1.7	Peroxidase superfamily protein
AT5G64110	6.801	1.665	-2.030	EC-1.11.1.7	Peroxidase superfamily protein
AT5G64120	55.197	27.423	-1.009	EC-1.11.1.7	PRX71; A cell wall bound peroxidase that is induced by hypo-osmolarity and is involved in the lignification of cell walls.
AT5G66390	14.289	6.961	-1.037	EC-1.11.1.7	PRX72; peroxidase that is involved in lignin biosynthesis.
AT5G67400	14.606	5.960	-1.293	EC-1.11.1.7	RHS19; peroxidase activity, heme binding
AT5G66690	12.768	3.290	-1.956	EC-2.4.1.111	UGT72E2; UDP-Glycosyltransferase superfamily protein
Up-regulated genes					
AT1G61720	4.760	33.855	2.830	EC-1.3.1.77	BAN; anthocyanidin reductase
AT3G51240	34.812	75.059	1.108	EC-1.14.11.9	TT6, F3H; flavanone 3-hydroxylase
AT4G22880	10.361	25.357	1.291	EC-1.14.11.19	ANS, LDOX; leucoanthocyanidin dioxygenase
AT4G26220	19.581	43.845	1.163	EC-2.1.1.104	CCoAOMT7; Caffeoyl-coenzyme A O-methyltransferase
AT5G07990	11.107	33.287	1.583	EC-1.14.11.9	TT7; Flavonoid 3' hydroxylase activity
AT5G42800	8.307	26.940	1.697		DFR; dihydroflavonol 4-reductase
AT5G63600	10.517	3.343	-1.653	EC-1.14.11.23	FLS5; flavonol synthase 5

Moreover, DEGs encoding F3H/TT7 (AT5G07990), DFR/TT3 (AT5G42800), ANS/LDOX (AT4G22880), and BAN (AT1G61720), involved in the flavonoid biosynthetic pathway of *Arabidopsis*, were up-regulated in transgenic line L8 (Fig 7B and Table 2). As anthocyanins, flavonols and proanthocyanidins are synthesized through the flavonoid biosynthetic pathway [44–48], one would expect the amount of anthocyanins, flavonols, and proanthocyanidins to be reasonably increased in L8. We therefore quantitated the anthocyanin content and found that the siliques of transgenic line L8 had higher anthocyanin than did those of Col-0 (S3 Fig), indicating that flavonoid biosynthesis has been enhanced when *BnLATE* overexpressed in *Arabidopsis*.

### Phenotyping of *pBnLATE*::*BnLATE* transgenic lines

**Fig 8:**
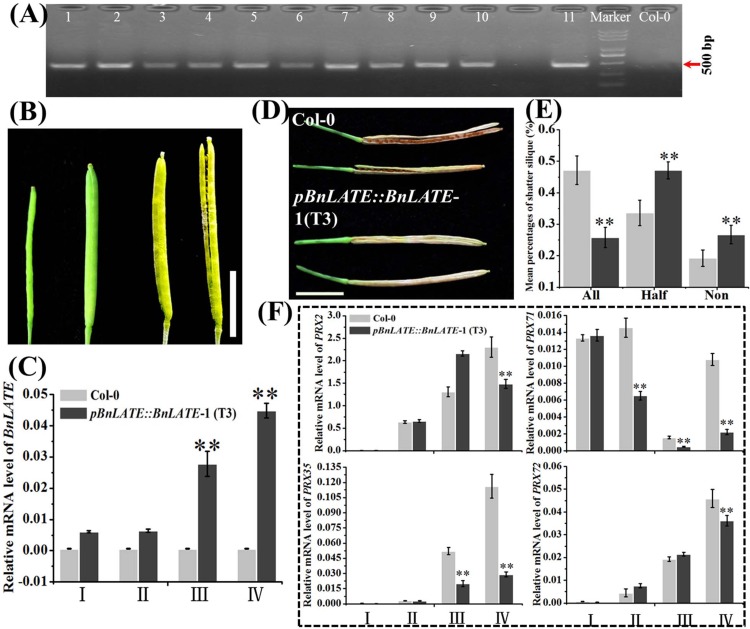
*BnLATE* expression variation and silique morphology of *pBnLATE*::*BnLATE* transgenic *Arabidopsis* lines. (A) Validation of target insert using specific PCR primers in 11 independent *pBnLATE*::*BnLATE Arabidopsis* lines (1–11). (B) Silique morphology at four developing stages 5 DAP (I), 10 DAP (II), 20 DAP (III), and 30 DAP (IV). Bar, 0.50 cm. (C) Expression pattern of *BnLATE* in silique of *pBnLATE*::*BnLATE transgenic* lines using Real-time PCR. Expression level of *BnLATE* was determined relative to that of the internal control *β-AtACTIN2* (AT3g18780), multiplied by 10. The experiment was performed in triplicate. (D) Representative yellowed siliques of Col-0 and *pBnLATE*::*BnLATE* transgenic lines. Bar, 0.50 cm. (E) Average percentage of high-, middle- and low-shattering-resistance siliques of *pBnLATE*::*BnLATE* transgenic lines, three biological replicates with 30 siliques each. Silique of Col-0 shows shattering-resistance. Asterisks above the columns indicate the significant difference vs. Col-0. * p< 0.05, ** p< 0.01. (F) Expression pattern of four representative peroxidase genes (*PRX2*, *PRX35*, *PRX71*, and *PRX72*) in siliques of *pBnLATE*::*BnLATE-1* (T3) and Col-0 by real-time PCR. Expression level was determined relative to that of the internal control *β-AtACTIN2* (AT3G18780). The experiment was performed in triplicate. Significance was defined with Student’s *t*-test. Asterisks above the columns indicate the significant difference compared with Col-0 and EV. * p< 0.05, ** P< 0.01.

In order to further confirm the effect of *BnLATE* on lignin biosynthesis in yellow silique wall, an expression vector of *BnLATE* driven by its own promoter was constructed. Following transformation, selection with hygromycin B and confirmation with PCR, 11 independent homogenous transgenic lines were reserved. For ease of operation, we chose one from the 11 transgenic lines and assigned as *pBnLATE*::*BnLATE*-1 (T_3_) for further investigation (Fig 8A).

**Fig 9:**
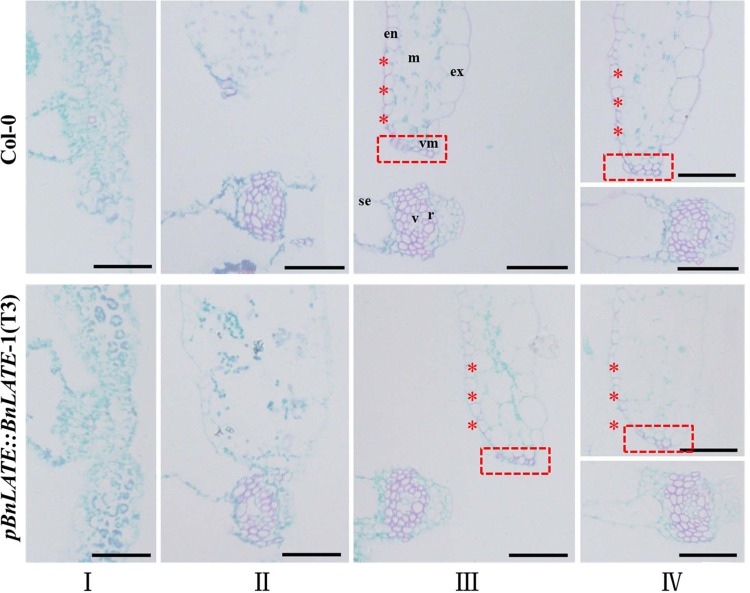
Histological observation on silique of *pBnLATE*::*BnLATE* transgenic lines. One representative result of five independent experiments is shown. Cross-sections of silique replum and carpel of *pBnLATE*::*BnLATE* and Col-0 at stages I, II, III and IV were performed and stained with safranin/fast green. In contrast to Col-0, no obvious staining color (red) was observed in the carpel of *pBnLATE*::*BnLATE* at stages III and IV, and lignification cells in the dehiscence zone was apparently less as well. Abbreviations: en, endocarp; ex, exocarp; m, mesocarp; r, replum; se, septum; v, vascular bundle; vm, valve margin. Bars, 50 μm.

The expression pattern of *BnLATE* in siliques of *pBnLATE*::*BnLATE*-1 and Col-0 was canvased using qRT-PCR at 5 DAP (I), 10 DAP (II), 20 DAP (III), and 30 DAP (IV) (Fig 8B). Expression level of *BnLATE* in *pBnLATE*::*BnLATE*-1 line at stages III and IV were apparently higher than that at stages I and II (Fig 8C), and these observations further demonstrated the reliability of the *pBnLATE* activity detected in *pBnLATE*::*GUS* transgenic lines (Fig 3A). Moreover, expression pattern of four peroxidase genes (*PRX2*, *PRX35*, *PRX71*, and *PRX72*) in the siliques of *pBnLATE*::*BnLATE*-1 line and Col-0 at stages I, II, III, and IV were identified with qRT-PCR as well. Compared with Col-0, all four genes were selectively down-regulated at stages III or/and IV in *pBnLATE*::*BnLATE*-1 line (Fig 8F) as well as in *D35S*::*BnLATE* transgenic lines revealed by RNA-seq (Fig 7A and Table 2). Consequently, microscopic observation was performed on replum and carpel of *pBnLATE*::*BnLATE*-1 and Col-0 stained by safranin/fast green at stages I, II, III, and IV. In contrast to Col-0, no obvious staining color (red) was observed in the carpel of *pBnLATE*::*BnLATE*-1 at stages III and IV, and lignification cells in the dehiscence zone was apparently lessened as well (Fig 9). Meanwhile, mature siliques of *pBnLATE*::*BnLATE*-1 line exhibited enhanced silique shattering resistance vs. Col-0 (Fig 8D and 8E). These observations further demonstrated that higher expressing *BnLATE* could strongly decrease lignin biosynthesis in yellowing silique walls through depressing genes encoding peroxidases, resulting in enhanced silique shattering resistance.

## Discussion

*LATE* was a member of the C_2_H_2_-type zinc-finger protein family. Several members of this family displayed essential functional roles in developmental or signaling processes. For example, SUPERMAN, RABBIT EARS, and KNUCKLES displayed crucial functions during flower development [49–51]. In this study, the function of *BnLATE*, identified with microarray screening on inflorescences from *B*. *napus* lines ZS11 and 73290, was investigated [34]. *BnLATE* was specifically expressed in the yellowing silique walls of *BnLATE* transgenic Arabidopsis (Fig 3A), and heterogenous overexpression of *BnLATE* in *Arabidopsis* had reduced lignification degree in the valve, replum, and valve margin cells of the silique wall, resulting in enhanced silique shattering resistance in contrast to the controls Col-0 and EV at 15 DAP (Figs 3C and 4 and Table 1). Endogenous *BnLATE* was remarkably expressed in silique walls of 73290 at 48 DAP (Fig 5A), that was similar to the pattern of *pBnLATE*::*GUS* activity in *Arabidopsis* (Fig 3A). Furthermore, the lignin content in the replum, valve margin, and valve layer from 12 to 36 DAP increased with greater gradient in 73290, but the lignin abundance declined slightly at 48 DAP vs. at 36 DAP (Fig 5B). These results further proved by lignin content variation in the silique walls of 73290 measured at 12, 24, 36, 48 and 60 DAP (Fig 5C). The shattering resistance index (SRI) of 73290 was high as 0.78 and 0.75 at 252 and at 280 revolutions per minute (rpm), respectively (Fig 5D). At the same time, we constructed a *pBnLATE*::*BnLATE* construct containing a 1358-bp fragment from ZS11, which including the complete CDS of *BnLATE* (513 bp) and its putative promoter (845 bp), was generated and transformed into *Arabidopsis* Col-0. The expression pattern of *BnLATE* in siliques of Col-0 and *pBnLATE*::*BnLATE*-1 (Fig 8B) indicated that the higher level of *BnLATE* expression was detectable in yellowing silique (III, IV) (Fig 8C), it further confirmed the reliability of the *pBnLATE*::*GUS* expression pattern observed in *Arabidopsis* (Fig 3A). In contrast to Col-0, no staining (red) was observed in carpel of *pBnLATE*::*BnLATE*-1 at stages III and IV, and lignification cells in the dehiscence zone were obviously reduced as well (Fig 9). Meanwhile, we had also found enhanced shattering resistance in mature siliques of *pBnLATE*::*BnLATE*-1 vs. the Col-0 (Fig 8D and 8E). Similar phenotypic variations happened upon the siliques both *D35S*::*BnLATE* and *pBnLATE*::*BnLATE* heterogenous transgenic *Arabidopsis* lines (Figs 3, 4, 8C, 8D, 8E and Table 1), and endogenous rapeseed varieties 73290 (Fig 5), indicating that silique shattering resistance increased along with the reduction of lignification extent due to higher expression of *BnLATE* in *B*. *napus*.

Using microarray analysis, 16 out of 27 genes involved in monolignol biosynthesis pathway in the Brassica cultivars were moderately to highly down-regulated in valve and DZ of *B*. *juncea* compared to *B*. *napus*. Light microscopy revealed a smaller non-lignified separation layer in relatively shatter-resistant *B*. *juncea* relative to *B*. *napus* [20]. In this study, we observed the same phenomenon in the heterogenous *D35S*::*BnLATE* and *pBnLATE*::*BnLATE* transgenic *Arabidopsis* lines (Figs 6, 7 and 8F). Nineteen genes encoding peroxidase (EC-1.11.1.7), two encoding UDP-glucosyl transferase (EC-2.4.1.111), and two encoding β-glucosidase (EC-3.2.1.21), which involveed in phenylpropanoid pathway, were down-regulated in the silique of transgenic *Arabidopsis* line L8 (Table 2). Phenylalanine was converted to yield monolignols (*p*-hydroxyphenyl (H), guaiacyl (G), and syringyl (S) units) through chemical reactions [9, 15, 38–41] (Fig 7C), monolignols were polymerized in a cellulose and hemicellulose matrix outside the cell by class III peroxidases (plant peroxidases; EC 1.11.1.7) [38]. The peroxidase homologs PRX2, PRXR9, PRX71, and PRX72 had been verified to oxidize not only monolignols efficiently but also lignin oligomers directly [15, 38–41] (Table 2). The histochemical study also revealed a low content in syringyl units and a decrease in amount of lignin in the *prx2*, *prx71*, and *prx72* mutant plants compared with the control [38, 41]. These results were highly consistent with our observations of lignin content variation in the siliques of *D35S*::*BnLATE* transgenic *Arabidopsis* lines (L21, L7 and L8) vs. the controls Col-0 and EV (Fig 4). Furthermore, the peroxidase genes (*prx2*, *prx35*, *prx71*, and *prx72*) expression significantly reduced in the *pBnLATE*::*BnLATE* transgenic lines during yellowing silique (III, IV) compared with that of Col-0 (Fig 8F). These observations suggest that *BnLATE* was a negative regulator of lignin biosynthesis in the yellowing silique walls through restraining the polymerization of monolignols and lignin, and promotes silique shattering resistance in *B*. *napus* attributive to higher expression.

*AtLATE* in *Arabidopsis* functions a s transcription activators, resulting in repression of flowering [37]. In the meristem, *AtLATE* interferes the establishment and maintenance of floral meristems. In the vasculature, ectopic *AtLATE* expression leads to a down-regulation of key elements to photoperiodic flowering response. However, novel phenotypic variation were observed on overexpressed *BnLATE* transgenic *Arabidopsis* lines. Contrasting to the controls Col-0 and EV at 15 DAPs, *D35S*::*BnLATE* transgenic *Arabidopsis* lines exhibited enhanced pod shattering-resistance (Fig 3C and Table 1), and apparently reduced lignification degree of silique walls in the valve, replum, and valve margin cells in transgenic lines (L21, L7, L8) (Fig 4). Because only 76% identity upon amino acid sequence was detectable between BnLATE and AtLATE, therefore those phenotypic discrepancies might be attributive to the sequence differences. Therefore, 3D models of proteins AtLATE and BnLATE were predicted using an online tool Phyre2 (http://www.sbg.bio.ic.ac.uk/phyre2) [52]. The results showed that the main difference between AtLATE and BnLATE proteins was caused by secondary structure (S4 Fig). Within the different region, two β-pleated sheets (green colour) in AtLATE protein but three α-helixs (white colour) in BnLATE protein were formed (S4B and S4C Fig), causing different 3D models in AtLATE and BnLATE (S4D Fig). As we know, appropriate space structure of the protein was a crucial factor to specific biological function, it is reasonable to deduce that BnLATE could conduct a specific phenotypic variation in *B*. *napus*.

Recent research had demonstrated that several genes tightly linked to silique shattering resistance. For example, mutants of *SHP1*, *SHP2*, *ALC*, *IND*, *FUL*, *RPL* and *SHP* showed lignification reduction in the dehiscence zone, replum and carpel in which lacked the small cells of the dehiscence zone and the adjacent lignified cell layers. Even conserved partial valve tissues developed into valve margin-like tissues (including lignified and dehiscence zone-like cell types), or formed into narrow files of replum cells that resemble those in the valve margin. Furthermore, seed development was reduced through valve elongation restrained after fertilization, especially in the basal part of the fruit due to a lack of coordinated growth of the fruit tissues, and resulted in yield failure [10, 12, 17–19]. In this study, overexpression of *BnLATE* resulted in less lignification in the replum, valve layer and dehiscence zone of yellowing silique in *B*. *napus*, as well as enhanced silique shattering resistance (Figs 3, 4, 8 and 9). However, no significant differences were observed in the structure of the dehiscence zone, replum, carpel, and valve margin cells (Figs 4B, 4C and 9), and no any other phenotypic variations occurred during silique and seed development. Our results provide novel insight into the mechanism underlying silique shattering resistance in *B*. *napus*, and form the basis for developing rapeseed cultivars with enhanced shattering resistance and thus increased yield.

## Supporting Information

S1 Fig Predicted functional domains of *BnLATE* promoter. (TIF)

S2 Fig An overview of GUS activity of *pBnLATE*::*GUS* transgenic *Arabidopsis* throughout the life cycle. Vegetative meristems were marked for the red arrows.
(TIF)

S3 Fig Total anthocyanin content in the siliques of wild-type Col-0 and *D35S*::*BnLATE* transgenic *Arabidopsis* line L8. The experiment was performed in biological triplicate and technical triplicate, and results represent the mean ± S.D of the three biological replicates. Significance was determined using Student’s *t*-test. Asterisks indicate significant differences between Col-0 and EV. * p< 0.05, ** p< 0.01.
(TIFF)

S4 Fig Prediction of AtLATE and BnLATE protein structure using PHYRE2. (A) Cartoon models of AtLATE and BnLATE protein. Image coloured by rainbow N to C terminus. AtLATE, and BnLATE models from left to right.
(B) Cartoon models of AtLATE and BnLATE protein. 76% identity amino acid sequences were indicated in red colour, and the main differences amino acid sequence (E) were indicated in green and white colour at AtLATE and BnLATE protein, respectively. AtLATE, and BnLATE models from left to right.
(C) Merged cartoon models of (B).
(D) Spheres models of AtLATE and BnLATE protein. 76% identity amino acid sequence was indicated in red colour, and the main differences amino acid sequence (E) were indicated in green and white colour at AtLATE and BnLATE protein, respectively. AtLATE, BnLATE, and merged models from up to down.
(E) Amino acid sequence alignment of AtLATE and BnLATE. Dotted black line boxes showed that the main differences amino acid sequence (80–140 AA).
(TIF)

S1 Table An overview of differentially expressed genes in the siliques of the D35S-BnLATE transgenic line L8 and wild-type Col-0 plants. (XLSX)

S2 Table Differentially expressed genes mapped by KEGG pathway mapping. (XLSX)

S3 Table Primers of reference and target genes used for real-time PCR. (XLSX)
