# Supplementary figures and images for: BnLATE, a Cys2/His2-Type Zinc-Finger Protein, Enhances Silique Shattering Resistance by Negatively Regulating Lignin Accumulation in the Silique Walls of Brassica napus
Source: PLoS One. 2017 Jan 12;12(1):e0168046. doi: 10.1371/journal.pone.0168046 (PMC5231383; doi:10.1371/journal.pone.0168046)

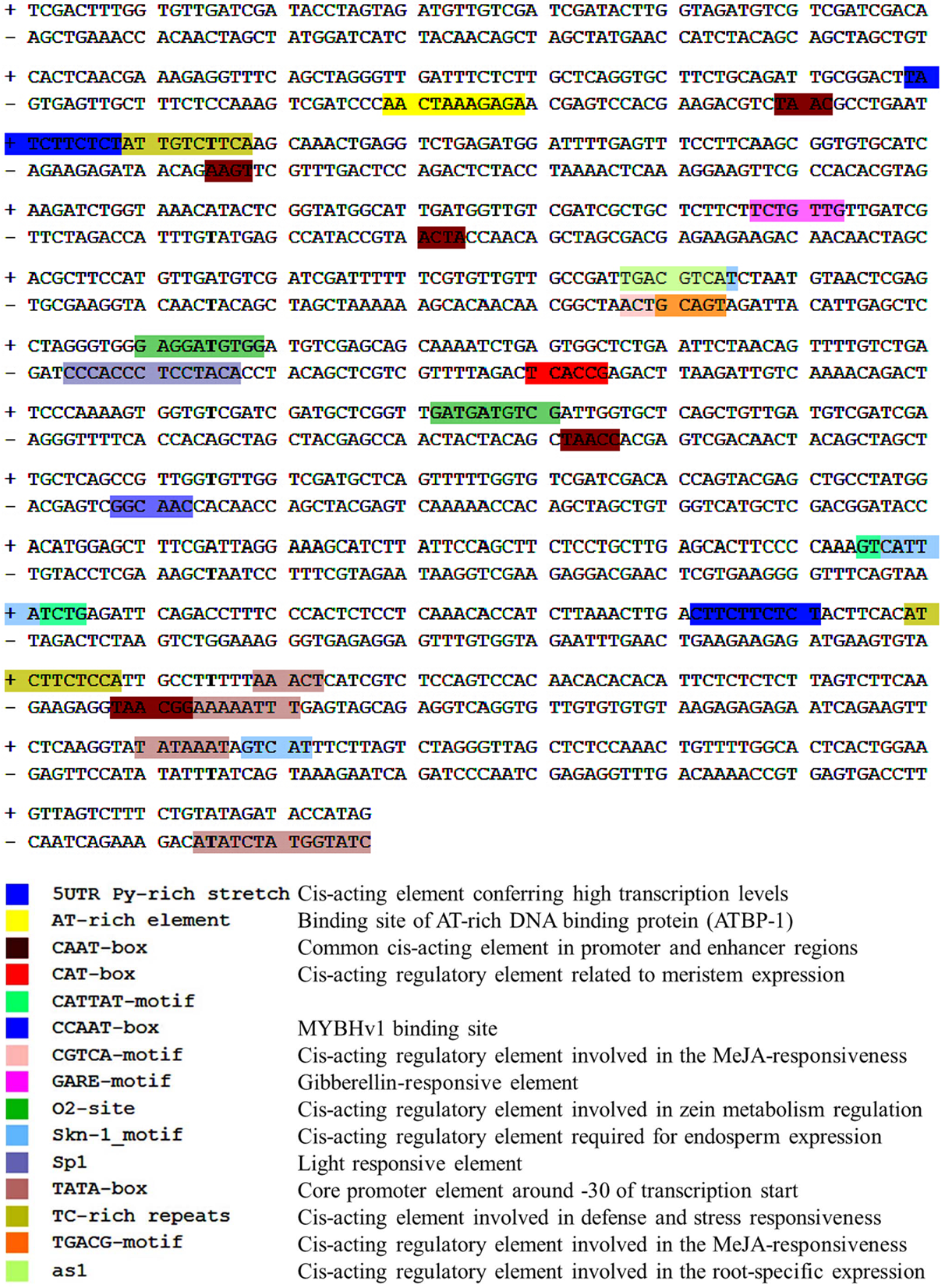

Supplement: S1 Fig — (TIF) [file pone.0168046.s001.tif]

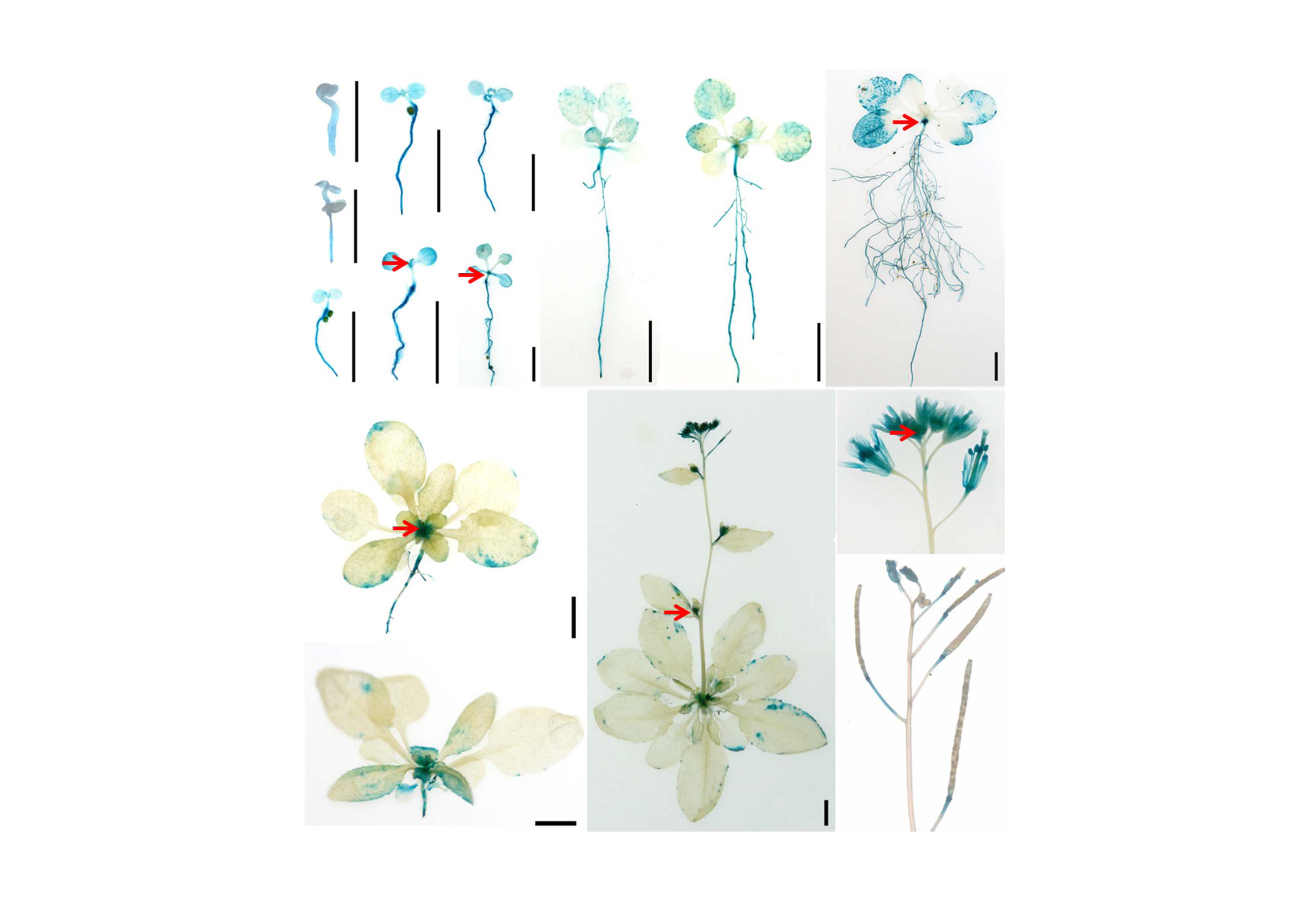

Supplement: S2 Fig — Vegetative meristems were marked for the red arrows. (TIF) [file pone.0168046.s002.tif]

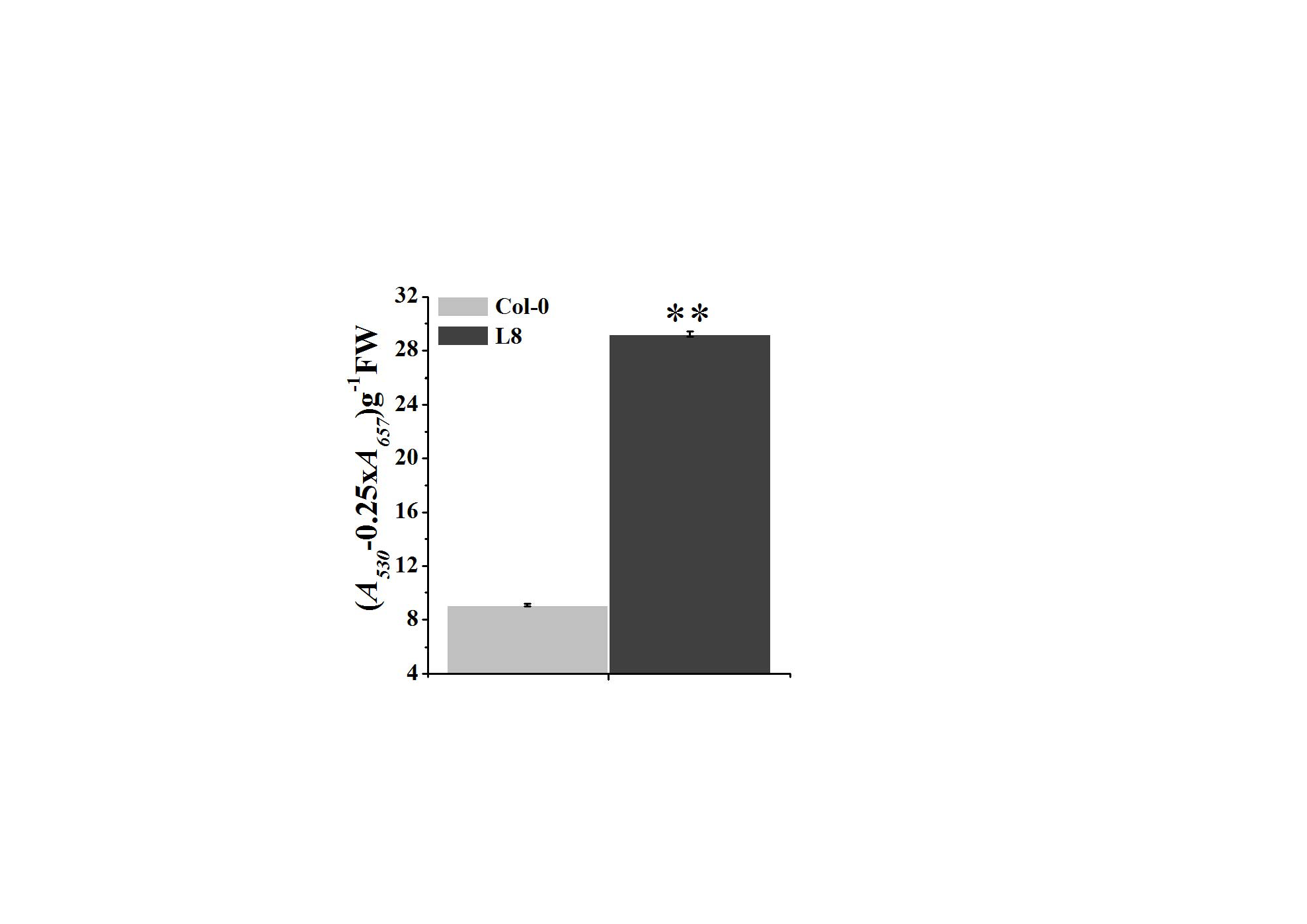

Supplement: S3 Fig — The experiment was performed in biological triplicate and technical triplicate, and results represent the mean ± S.D of the three biological replicates. Significance was determined using Student’s t-test. Asterisks indicate significant differences between Col-0 and EV. * p< 0.05, ** p< 0.01. (TIFF) [file pone.0168046.s003.tiff]

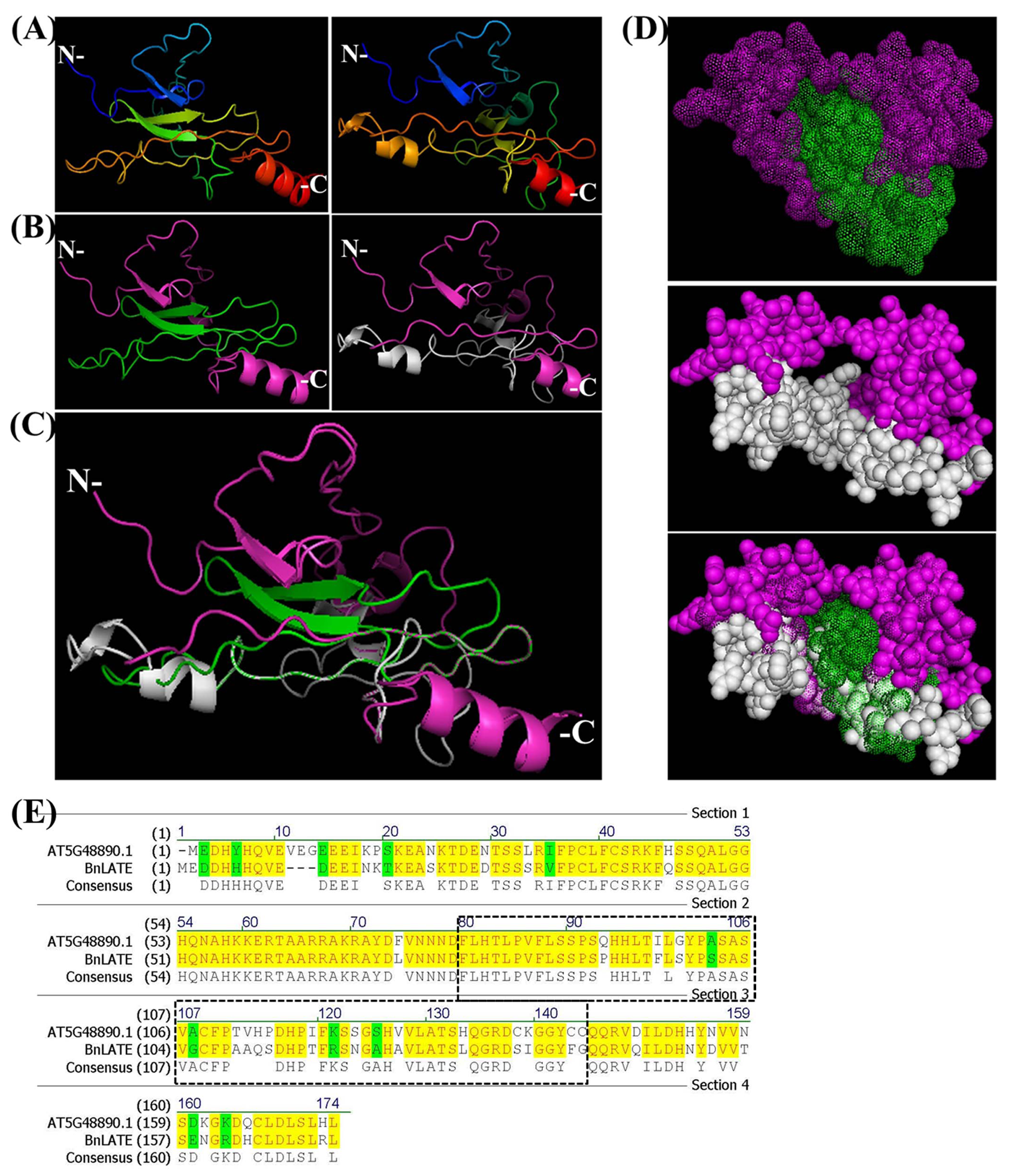

Supplement: S4 Fig — (A) Cartoon models of AtLATE and BnLATE protein. Image coloured by rainbow N to C terminus. AtLATE, and BnLATE models from left to right. (B) Cartoon models of AtLATE and BnLATE protein. 76% identity amino acid sequences were indicated in red colour, and the main differences amino acid sequence (E) were indicated in green and white colour at AtLATE and BnLATE protein, respectively. AtLATE, and BnLATE models from left to right. (C) Merged cartoon models of (B). (D) Spheres models of AtLATE and BnLATE protein. 76% identity amino acid sequence was indicated in red colour, and the main differences amino acid sequence (E) were indicated in green and white colour at AtLATE and BnLATE protein, respectively. AtLATE, BnLATE, and merged models from up to down. (E) Amino acid sequence alignment of AtLATE and BnLATE. Dotted black line boxes showed that the main differences amino acid sequence (80–140 AA). (TIF) [file pone.0168046.s004.tif]
